# New Clonal Strain of *Candida auris*, Delhi, India

**DOI:** 10.3201/eid1910.130393

**Published:** 2013-10

**Authors:** Anuradha Chowdhary, Cheshta Sharma, Shalini Duggal, Kshitij Agarwal, Anupam Prakash, Pradeep Kumar Singh, Sarika Jain, Shallu Kathuria, Harbans S. Randhawa, Ferry Hagen, Jacques F. Meis

**Affiliations:** Author affiliations: Vallabhbhai Patel Chest Institute, Delhi, India (A. Chowdhary, C. Sharma, K. Agarwal, A. Prakash, P.K. Singh, S. Kathuria, H.S. Randhawa); B.L. Kapur Memorial Hospital, New Delhi, India (S. Duggal, S. Jain); Canisius-Wilhelmina Hospital, Nijmegen, the Netherlands (F. Hagen, J.F. Meis); and Radboud University Nijmegen Medical Centre, Nijmegen (J.F. Meis)

**Keywords:** Candida auris, fungemia, M13 fingerprinting, AFLP, antifungal susceptibility, India, fungi, parasitic diseases

## Abstract

A new clonal strain of *Candida auris* is an emerging etiologic agent of fungemia in Delhi, India. In 12 patients in 2 hospitals, it was resistant to fluconazole and genotypically distinct from isolates from South Korea and Japan, as revealed by M13 and amplified fragment length polymorphism typing.

In 2009, yeast isolates from the external ear canal of a Japanese patient were identified as a new species, *Candida auris*, on the basis of sequence analysis of the nuclear rRNA gene. The species is phylogenetically related to *C. haemulonii* (*1*). In the same year, 15 isolates of *C. auris* were reported from otitis media patients in South Korea (*2*). Genotyping of these isolates revealed a clonal origin (*3*). In addition, in 3 patients, persistent fungemia caused by *C. auris* was resistant to fluconazole; 2 of these patients died (*4*). Fungemia caused by *C. auris* seems to be extremely rare, however. We report a series of fungemia cases caused by a new clonal strain of this emerging pathogen, involving 12 inpatients from 2 hospitals in Delhi, India.

## The Study

We characterized 12 bloodstream *C. auris* isolates, originating from an equal number of patients, collected during 2009–2011 at 2 hospitals (a tertiary care general hospital and a pediatric center) in Delhi, India. The controls included reference strains of *C. auris* from South Korea (CBS 12372, CBS 12373) and Japan (JCM15448, DSMZ21092), *C. haemulonii* (CBS 7801–7802, CBS 5149**,** CBS 5150), *C. pseudohaemulonii* (KCTC 1787, CBS 10004, JCM 12453), and *C. duobushaemulonii* (CBS 7798–7800, CBS 9754).

The yeast isolates were oval without pseudohyphae and germ tube formation. They appeared pink on CHROMagar Candida medium (Difco, Becton Dickinson, Baltimore, MD, USA) and grew at 37°C and 42°C. VITEK2 (bioMérieux, Marcy I’Etoile, France) misidentified 10 isolates as *C. haemulonii* and 2 as *C. famata*. Similarly, API20C (bioMérieux) also misidentified them as *C. sake*. *C. auris* isolates from India assimilated *N*-acetylglucosamine (NAG) in contrast to the isolates from Japan and South Korea.

Molecular identification was done by sequencing internal transcribed spacer (ITS) and D1/D2 regions (*5*–*7*). ITS sequences (GenBank accession nos. KC692039–KC692050) of our isolates showed 100% homology with an unrelated *C. auris* isolate (GenBank accession no. HE797773) and 98% identity with isolates from Japan and South Korea (GenBank accession nos. AB375772 and EU884189). Also, large ribosomal subunit sequences (GenBank accession nos. KC692053–KC692064) of the India isolates showed 100% homology with an unrelated *C. auris* isolate (GenBank accession no. HE797774) and 98%–100% identity with isolates from Japan and South Korea (accession nos. AB375773 and EU881960). ITS and large ribosomal subunit sequences of the isolates in our study showed 85%–98% similarity with the closest *C. haemulonii* complex species. These isolates have been deposited at the CBS-KNAW Fungal Biodiversity Centre, Utrecht, the Netherlands (accession nos. CBS 12766–12777).

**Figure 1:**
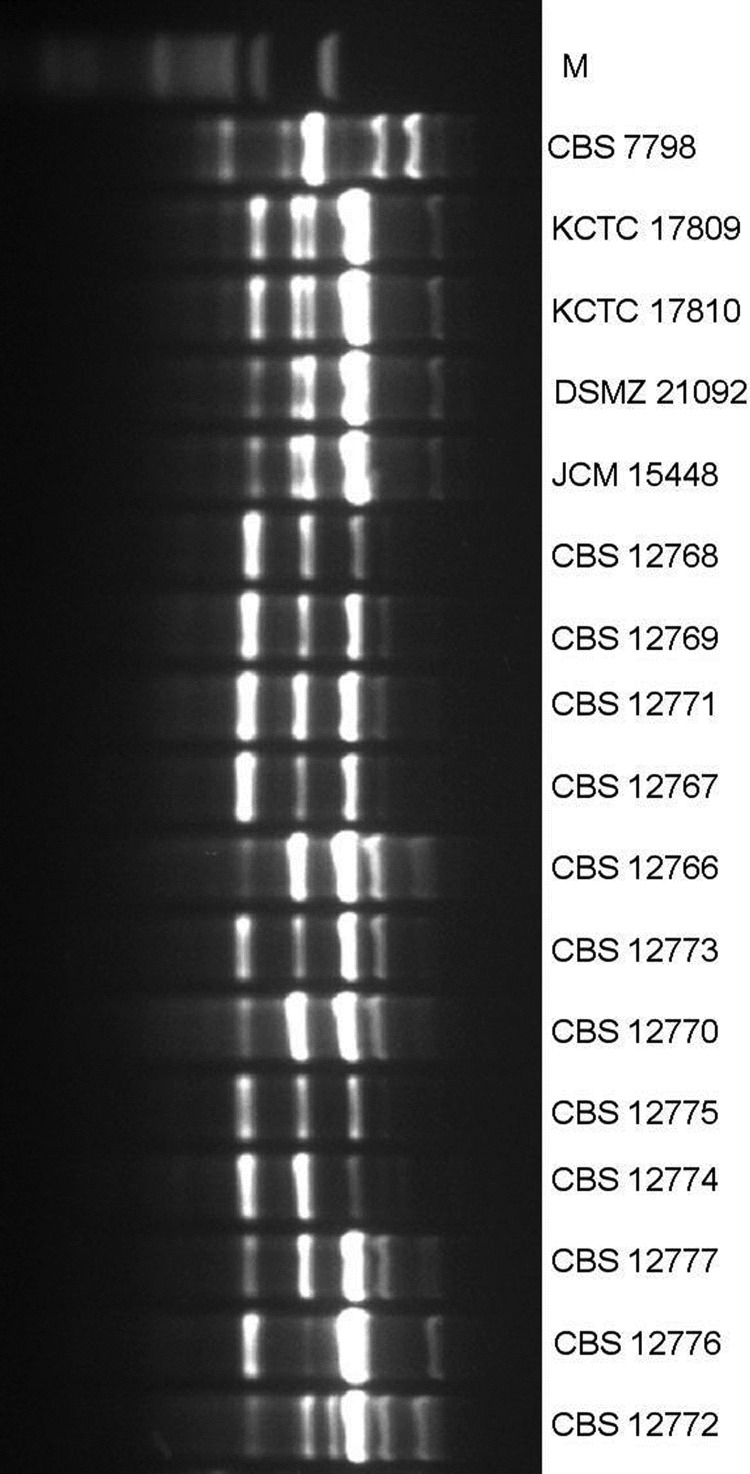
M13 PCR fingerprinting of *Candida auris* isolates. Lane 1, marker 50-bp ladder (New England BioLabs, Evry, France); lane 2, *C. duobushaemulonii* reference isolate (CBS 7798); lanes 3–6, *C. auris* isolates from South Korea (KCTC 17809 and KCTC 17810), Japan (DSMZ 21092 and JCM 15448), and reference isolates; and lane 7–18, 12 test isolates from India.

**Figure 2:**
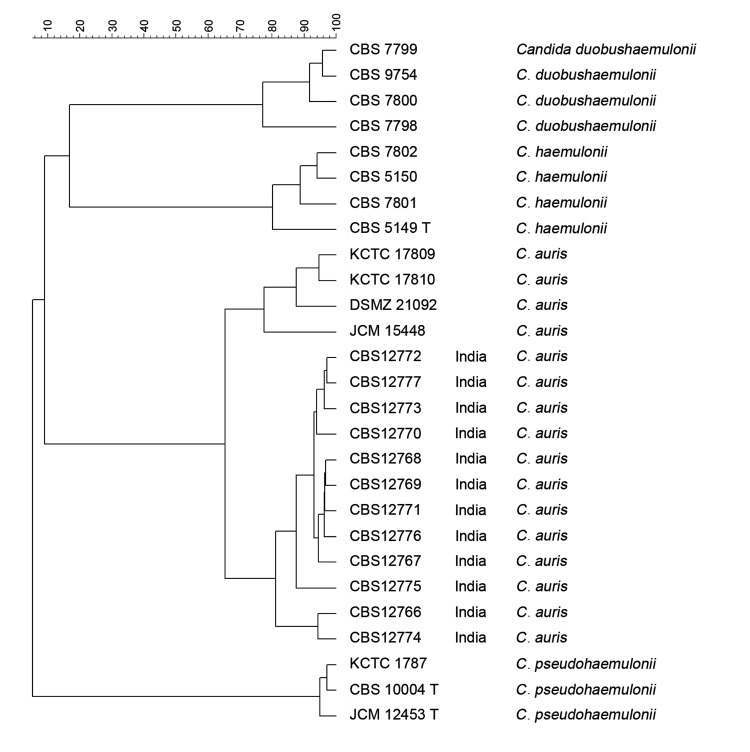
Dendrogram of amplified fragment length polymorphism analysis of *Candida auris* isolates from India, Japan, and South Korea and members of *C. haemulonii* complex. It was constructed by using UPGMA (unweighted pair group method with averages) in combination with the Pearson correlation coefficient and was restricted to fragments of 60–400 bp. Scale bar indicates the percentage similarity.

M13 PCR fingerprinting analysis was performed as described (*8*,*9*). The isolates from India had a distinct banding pattern in relation to isolates from Japan and South Korea. Except for 2 isolates (CBS 12772, CBS 12776), the *C. auris* isolates showed an identical banding pattern, suggesting a single genotype (Figure 1). The isolates’ genotypic diversity was also determined by using amplified fragment length polymorphism **(**AFLP) fingerprint analysis (*10*). In a dendrogram in which standard Pearson and UPGMA (unweighted pair group method with averages) settings were used, *C. auris* isolates clustered separately from *C. haemulonii*, *C. duobushaemulonii*, and *C. pseudohaemulonii*. The *C. auris* isolates from India showed a clonal origin and were genotypically distinct from the isolates from Japan and South Korea (Figure 2).

Antifungal susceptibility testing was done by using microbroth dilution according to Clinical Laboratory Standards Institute protocols (*11*). All isolates showed high geometric mean MICs of fluconazole (Pfizer, Groton, CT, USA) (28.5 mg/L), whereas isavuconazole (Basilea Pharmaceutica, Basel, Switzerland [now Astellas]), posaconazole (Schering-Plough, Kenilworth, NJ, USA [now Merck]), itraconazole (Lee Pharma, Hyderabad, India), and voriconazole (Pfizer) exhibited highly potent activity. Amphotericin B (Sigma-Aldrich, St. Louis, MO, USA), flucytosine (Sigma-Aldrich), and the 3 echinocandins also showed excellent activity (Table 1).

**Table 1:** Results of In vitro antifungal susceptibility testing of 12 *Candida auris* isolates originating from fungemia patients in 2 hospitals, Delhi, India, 2009–2011

MIC, mg/L*	Drug†									
	AMB	FLU	ITC	VRC	ISA	POS	FC	CAS	MFG	AFG
MIC_50_	0.25	32	0.125	0.5	0.125	0.125	0.125	0.25	0.06	0.25
MIC_90_	0.5	64	0.25	1	0.25	0.25	0.125	0.25	0.125	0.5
GM	0.33	28.5	0.15	0.39	0.10	0.12	0.125	0.23	0.072	0.26
Range	0.25–1	16–64	0.125–0.25	0.125–1	<0.015–0.25	0.06–0.25	0.125	0.125–0.25	0.06–0.125	0.125–0.5

*MIC_50_, 50% minimum inhibitory concentration; MIC_90_, 90% minimum inhibitory concentration; GM, geometric mean of MICs. †AMB, amphotericin B; FLU, fluconazole; ITC, itraconazole; VRC, voriconazole; ISA, isavuconazole; POS, posaconazole; FC, flucytosine; CAS, caspofungin; MFG, micafungin; AFG, anidulafungin.

Clinical features of the infections and response to antifungal therapy of the 12 patients with fungemia are summarized in the online Technical Appendix Table (wwwnc.cdc.gov/EID/article/19/10/13-0393-Techapp1.pdf). Each patient had at least 3 predisposing risk factors. Seven had immunosuppressive conditions: 6 (50%) had diabetes mellitus, 5 (42%) had chronic kidney disease, 2 (17%) had undergone cancer chemotherapy, and 1 (8%) was infected with HIV. Of the 5 children, 3 were infants of low birthweight, 1 neonate had late onset sepsis, and one 10-year-old child had acute lymphoblastic leukemia. Having an indwelling urinary catheter was the predominant risk factor for 83% of patients, followed by receiving broad-spectrum antimicrobial drugs (75%), spending time in an intensive care unit (58%), having neutropenia (50%), and having a central venous catheter (CVC) (42%). Notably, 7 (58%) patients had breakthrough fungemia while receiving fluconazole (1–3 weeks), and persistent candidemia developed in 9 (75%) patients. In 8 of these 9 patients, candidemia cleared after antifungal therapy in 1–3 weeks. For the remaining 3 patients, blood culture reports were available post mortem. *C. auris* represented 5% of the annual candidemia cases in a pediatric hospital and 30% of annual candidemia cases in a tertiary care general hospital. Overall mortality rates as high as 50% were noted; 30% of deaths were attributed to critical illness.

## Conclusions

This study documents the emergence of a new clonal strain of *C. auris* as an etiologic agent of candidemia in India. Fungemia caused by *C. auris* is extremely rare, identified only by sequencing. Because diagnostic laboratories do not undertake molecular identification routinely, fungemia is likely to be much more prevalent than published reports indicate. Growth at 40°C may differentiate *C. auris* isolates, routinely misidentified as *C. haemulonii* by VITEK. *C. haemulonii* does not grow at 40°C, as does *C. auris* (*12*). Sequencing may confirm their identity.

Although Lee et al. proposed negative assimilation of NAG for differentiating *C. auris* from *C. pseudohaemulonii*, this proposal seems invalid because all isolates from India assimilated NAG (*4*). The phenotypic divergence of these isolates was also supported by M13 and AFLP typing. Notably, *C. auris* isolates from 2 hospitals in Delhi were clonal, suggesting interhospital transmission. Transmission of the same genotype was observed in intensive care, surgical, medical, oncologic, neonatal, and pediatric wards, which were mutually exclusive with respect to health care personnel. This finding is analogous to that of the clonal transmission of *C. auris* among 3 hospitals in South Korea (*2*). Prevalence of a unique clonal strain of *C. auris* in 2 hospitals in Delhi, 13.5 km apart, underscores the need for more comprehensive studies to determine potential occurrence of other endemic clones in various regions of India.

Because most (83%) of the patients in our study had an indwelling urinary catheter, the source of fungemia could be urogenital colonization. However, no urine samples were cultured. Five (42%) patients had a CVC in situ, but none received total parenteral nutrition. Although *Candida* spp. adhere to intravascular catheters, providing a potential nidus for infection, a previous study showed that *C. auris* does not form a biofilm (*3*). Catheter tip cultures were negative for 3 of the 5 patients who had a CVC. Although 57% of patients admitted to critical care units died, the role of *C. auris* candidemia in fatal cases could not be ascertained because no comparison was available with a population that did not have candidemia.

*C. auris* assumes a greater clinical significance for the successful management of candidemia because all the reported isolates, including the new isolates, were fluconazole resistant (*2*,*4*). Most patients in this study had persistent candidemia, and mortality rates were as high as 33%.

In conclusion, *C. auris* is an emerging yeast pathogen that is underreported because it is misidentified in routine diagnostic laboratories. The resistance of *C. auris* to fluconazole is particularly worrisome.

Technical Appendix Clinical evaluation of 12 *Candida auris* fungemia patients investigated from 2 hospitals, Delhi, India, 2009–2011.

## Technical Appendix

**Table:** Technical Appendix Table. Clinical evaluation of 12 *Candida auris* fungemia patients investigated from 2 hospitals, Delhi, India, 2009–2011

Characteristic	Case-patient no.											
	1	2	3	4	5	6	7	8	9	10	11	12
Age/sex	3 d/F	10 d/F	28 d/F	45 d/F	10 y/M	45 y/M	47 y/F	59 y/M	60 y/F	65 y/F	67 y/M	74 y/M
Diagnosis	PT, TEF, ICH sepsis	PT, ELBW, sepsis, DIC	Pneumonia, late onset sepsis	Meningitis, septic shock, PDA, ASD, fetal distress, IMV	ALL, CKD	Decompensated alcoholic liver cirrhosis, CKD, sepsis	DM, ESRD	HIV, cryptococcal meningitis, DM	ESRD, DM	Recurrence of ovarian carcinoma, DM	COPD exacerbation sepsis, DM, ESRD	Jejunal perforation, peritonitis, septicemia, MODS, DM
Risk factor												
Immunosuppression	+	+	–	+	+	+	+	+	+	+	+	+
Neutropenia (<10^9^ cells/L)	+	+	+	-	+	–	–	+	–	+	–	–
CVC	–	+	+	+	+	–	–	–	–	–	–	+
Broad-spectrum antibiotics	+	+	+	+	+	–	-	+	+	+	–	+
Parenteral nutrition	–	–	–	-	–	–	-	–	–	–	–	–
Surgery within 30 d	–	–	–	+	–	–	-	–	–	–	+	+
I ntensive care	+	+	+	+	+	+	-	–	–	–	–	+
Antifungals within 30 d	+	+	+	–	+	–	+	+	–	–	–	+
Concomitant bacteremia	+	+	+	+	–	–	-	+	–	–	–	+
Indwelling urinary catheter	+	+	+	+	+	–	+	+	–	+	+	+
Day of isolation	3	3	20	10	18	1	3	21	9	1	Day 10	Day 21
Therapy (dosage)*	CAS (loading dose of 70 mg, then 50 mg daily)	AMB (0.5 mg/kg BW)	AMB (0.5 mg/kg BW)	AMB (0.5 mg/kg BW)	No antifungal given	No antifungal given	AMB (0.5 mg/kg BW)	AMB (1 mg/kg BW)	CAS (loading dose of 70 mg, then 50 mg daily)	AMB (1 mg/kg BW)	FLU (400 mg OD)	CAS (loading dose of 70 mg, then 50 mg daily)
Duration of therapy	5 d	3 wk	3 wk	1 wk	Not given	Not given	2 wk	1 wk	2 wk	2 wk	4 wk	2 wk
Clearance of candidemia*	NA	10 d	10 d	7 d	NA	NA	Not achieved after 14 d	7 d	10 d	7 d	3 wk	10 d
Outcome	Died 8 d after admission	Discharge	Discharge	Discharge	Died 2 d after admission	Died on the day of admission	Died 17 d after admission	Died after 28 d	Discharged in a stable state	Discharged in a stable state	Discharged on 2 wk of FLU	Died 14 d after presentation

*PT, pre-term; TEF, tracheo-esophageal fistula; ICH, intracranial hemorrhage; ELBW, extremely low birth weight; DIC, disseminated intravascular coagulation; PDA, patent ductus arteriosus; ASD, atrial septal defect; IMV, invasive mechanical ventilation; ALL, acute lymphocytic leukemia; CKD, chronic kidney disease; DM, diabetes mellitus; ESRD, end-stage renal disease; COPD, chronic obstructive pulmonary disease; MODS, multi organ dysfunction syndrome; CVC, central venous catheter; CAS, caspofungin; AMB, amphotericin B, FLU, fluconzaole; BW, body weight; NA, not assessed. †Therapeutic failure was defined either as the persistence of *Candida* in the bloodstream despite adminstration of 3 d of antifungal therapy or as development of breakthrough candidemia while receiving antifungal agents for 3 d..
