# Supplementary material for: New Clonal Strain of Candida auris, Delhi, India: New Clonal Strain of Candida auris, Delhi, India
Source: Emerg Infect Dis. 2013 Oct;19(10):1670–3. doi: 10.3201/eid1910.130393 (PMC3810747; doi:10.3201/eid1910.130393)
Supplement: Technical Appendix — Clinical evaluation of 12 Candida auris fungemia patients investigated from 2 hospitals, Delhi, India, 2009–2011. [file 13-0393-Techapp-s1.pdf]

Article DOI: http://dx.doi.org/10.3201/eid1907.130393

New Clonal Strain of *Candida auris*, Delhi, India

Technical Appendix

Technical Appendix Table. Clinical evaluation of 12 *Candida auris* fungemia patients investigated from 2 hospitals, Delhi, India, 2009–2011

	Case-patient no.											
Characteristic	1	2	3	4	5	6	7	8	9	10	11	12
Age/sex	3 d/F	10 d/F	28 d/F	45 d/F	10 y/M	45 y/M	47 y/F	59 y/M	60 y/F	65 y/F	67 y/M	74 y/M
Diagnosis	PT, TEF, ICH sepsis	PT, ELBW, sepsis, DIC	Pneumonia, late onset sepsis	Meningitis, septic shock, PDA, ASD, fetal distress, IMV	ALL, CKD	Decompensated alcoholic liver cirrhosis, CKD, sepsis	DM, ESRD	HIV, cryptococcal meningitis, DM	ESRD, DM	Recurrence of ovarian carcinoma, DM	COPD exacerbation sepsis, DM, ESRD	Jejunal perforation, peritonitis, septicemia, MODS, DM
Risk factor												
Immunosuppression	+	+	–	+	+	+	+	+	+	+	+	+
Neutropenia (<10^9 cells/L)	+	+	+	-	+	–	–	+	–	+	–	–
CVC	–	+	+	+	+	–	–	–	–	–	–	+
Broad-spectrum antibiotics	+	+	+	+	+	–	-	+	+	+	–	+
Parenteral nutrition	–	–	–	-	–	–	-	–	–	–	–	–
Surgery within 30 d	–	–	–	+	–	–	-	–	–	–	+	+
Intensive care	+	+	+	+	+	+	-	–	–	–	–	+
Antifungals within 30 d	+	+	+	–	+	–	+	+	–	–	–	+
Concomitant bacteremia	+	+	+	+	–	–	-	+	–	–	–	+
Indwelling urinary catheter	+	+	+	+	+	–	+	+	–	+	+	+
Day of isolation	3	3	20	10	18	1	3	21	9	1	Day 10	Day 21
Therapy (dosage)*	CAS (loading dose of 70 mg, then 50 mg daily)	AMB (0.5 mg/kg BW)	AMB (0.5 mg/kg BW)	AMB (0.5 mg/kg BW)	No antifungal given	No antifungal given	AMB (0.5 mg/kg BW)	AMB (1 mg/kg BW)	CAS (loading dose of 70 mg, then 50 mg daily)	AMB (1 mg/kg BW)	FLU (400 mg OD)	CAS (loading dose of 70 mg, then 50 mg daily)
Duration of therapy	5 d	3 wk	3 wk	1 wk	Not given	Not given	2 wk	1 wk	2 wk	2 wk	4 wk	2 wk
Clearance of candidemia*	NA	10 d	10 d	7 d	NA	NA	Not achieved after 14 d	7 d	10 d	7 d	3 wk	10 d
Outcome	Died 8 d after admission	Discharge	Discharge	Discharge	Died 2 d after admission	Died on the day of admission	Died 17 d after admission	Died after 28 d	Discharged in a stable state	Discharged in a stable state	Discharged on 2 wk of FLU	Died 14 d after presentation

*PT, pre-term; TEF, tracheo-esophageal fistula; ICH, intracranial hemorrhage; ELBW, extremely low birth weight; DIC, disseminated intravascular coagulation; PDA, patent ductus arteriosus; ASD, atrial septal defect; IMV, invasive mechanical ventilation; ALL, acute lymphocytic leukemia; CKD, chronic kidney disease; DM, diabetes mellitus; ESRD, end-stage renal disease; COPD, chronic obstructive pulmonary disease; MODS, multi organ dysfunction syndrome; CVC, central venous catheter; CAS, caspofungin; AMB, amphotericin B, FLU, fluconzaole; BW, body weight; NA, not assessed.
†Therapeutic failure was defined either as the persistence of *Candida* in the bloodstream despite adminstration of 3 d of antifungal therapy or as development of breakthrough candidemia while receiving antifungal agents for 3 d..
